# Prophylactic cannabinoid administration blocks the development of paclitaxel-induced neuropathic nociception during analgesic treatment and following cessation of drug delivery

**DOI:** 10.1186/1744-8069-10-27

**Published:** 2014-04-18

**Authors:** Elizabeth J Rahn, Liting Deng, Ganesh A Thakur, Kiran Vemuri, Alexander M Zvonok, Yvonne Y Lai, Alexandros Makriyannis, Andrea G Hohmann

**Affiliations:** 1 Neuroscience and Behavior Program, Department of Psychology, University of Georgia, Athens, GA, USA; 2 Program in Neuroscience, Indiana University, Bloomington, IN, USA; 3 Interdisciplinary Biochemistry Graduate Program, Indiana University, Bloomington, IN, USA; 4 Gill Center for Biomolecular Science, Indiana University, Bloomington, IN, USA; 5 Center for Drug Discovery, Department of Pharmaceutial Sciences, Bouve College of Health Sciences, Northeastern University, Boston, MA, USA; 6 Department of Psychological and Brain Sciences, Indiana University, 702 N Walnut Grove Ave, Bloomington, IN 47405-2204, USA; 7 Present Address: Department of Neurobiology, University of Alabama at Birmingham, 1825 University Blvd., SHEL 1070C, Birmingham, AL 35294, USA

**Keywords:** Cannabinoid, CB_1_, CB_2_, Chemotherapy, Cold allodynia, Mechanical allodynia, Osmotic mini pump, Paclitaxel

## Abstract

**Background:**

Chemotherapeutic treatment results in chronic pain in an estimated 30-40 percent of patients. Limited and often ineffective treatments make the need for new therapeutics an urgent one. We compared the effects of prophylactic cannabinoids as a preventative strategy for suppressing development of paclitaxel-induced nociception. The mixed CB_1_/CB_2_ agonist WIN55,212-2 was compared with the cannabilactone CB_2_-selective agonist AM1710, administered subcutaneously (s.c.), via osmotic mini pumps before, during, and after paclitaxel treatment. Pharmacological specificity was assessed using CB_1_ (AM251) and CB_2_ (AM630) antagonists. The impact of chronic drug infusion on transcriptional regulation of mRNA markers of astrocytes (GFAP), microglia (CD11b) and cannabinoid receptors (CB_1_, CB_2_) was assessed in lumbar spinal cords of paclitaxel and vehicle-treated rats.

**Results:**

Both WIN55,212-2 and AM1710 blocked the development of paclitaxel-induced mechanical and cold allodynia; anti-allodynic efficacy persisted for approximately two to three weeks following cessation of drug delivery. WIN55,212-2 (0.1 and 0.5 mg/kg/day s.c.) suppressed the development of both paclitaxel-induced mechanical and cold allodynia. WIN55,212-2-mediated suppression of mechanical hypersensitivity was dominated by CB_1_ activation whereas suppression of cold allodynia was relatively insensitive to blockade by either CB_1_ (AM251; 3 mg/kg/day s.c.) or CB_2_ (AM630; 3 mg/kg/day s.c.) antagonists. AM1710 (0.032 and 3.2 mg/kg /day) suppressed development of mechanical allodynia whereas only the highest dose (3.2 mg/kg/day s.c.) suppressed cold allodynia. Anti-allodynic effects of AM1710 (3.2 mg/kg/day s.c.) were mediated by CB_2_. Anti-allodynic efficacy of AM1710 outlasted that produced by chronic WIN55,212-2 infusion. mRNA expression levels of the astrocytic marker GFAP was marginally increased by paclitaxel treatment whereas expression of the microglial marker CD11b was unchanged. Both WIN55,212-2 (0.5 mg/kg/day s.c.) and AM1710 (3.2 mg/kg/day s.c.) increased CB_1_ and CB_2_ mRNA expression in lumbar spinal cord of paclitaxel-treated rats in a manner blocked by AM630.

**Conclusions and implications:**

Cannabinoids block development of paclitaxel-induced neuropathy and protect against neuropathic allodynia following cessation of drug delivery. Chronic treatment with both mixed CB_1_/CB_2_ and CB_2_ selective cannabinoids increased mRNA expression of cannabinoid receptors (CB_1_, CB_2_) in a CB_2_-dependent fashion. Our results support the therapeutic potential of cannabinoids for suppressing chemotherapy-induced neuropathy in humans.

## Background

Cannabinoids attenuate or, in some cases, prevent pain associated with surgery [1], inflammation [2], internal organs [3], and neuropathies (for review see [4]). Neuropathic pain is associated with abnormal changes in the peripheral and/or central nervous system resulting in non-adaptive, chronic pain. Clinical manifestations of neuropathic pain are notoriously unresponsive to traditional analgesics. Chemotherapeutic treatment with antineoplastic agents, while effective at eliminating harmful malignancies, is also associated with severe side effects. Of these side effects, emesis, alopecia, and myelosuppression have received the spotlight; however, a new front runner has recently emerged. Neuropathic pain associated with chemotherapeutic treatment is dose-limiting and a major factor influencing discontinuation of treatment [5,6]. Chemotherapy-induced neuropathy is positively correlated with cumulative chemotherapeutic dose [7], and affected patients are more likely to experience other neuropathies [8]. An aging US population, coupled with diagnostic and medical advances in cancer treatment, means that more cancer survivors will be impacted by, and living longer with, chemotherapy-induced neuropathy. Thus, identification of prophylactic treatments that block development of chemotherapy-induced neuropathy represents an urgent medical need.

Chemotherapeutic agents are divided into three mechanistically distinct classes. These classes include the vinca alkaloids, platinum-derived agents, and taxanes. Taxanes (e.g., paclitaxel, docetaxel) produce antineoplastic effects by stabilizing microtubules through binding to β-tubulin, thereby disrupting normal cell mitosis and triggering the mitochondrial apoptosis pathway [9]. Paclitaxel is a preferred agent for treatment of ovarian, breast, and lung cancers; however, a high percentage of patients experience neuropathic pain – a type of pain poorly treated with available drugs [10]. Mechanisms underlying development of paclitaxel-induced neuropathy remain incompletely understood but may involve changes in glial activation [11,12].

Cannabinoid agonists suppress paclitaxel-induced neuropathic nociception in animal models through activation of both CB_1_[13] and CB_2_[14-16] cannabinoid receptor subtypes. Our laboratory first demonstrated CB_2_ receptor-mediated suppression of neuropathic allodynia induced by chemotherapeutic treatment with vincristine [17], paclitaxel [14,18], and cisplatin [15]. Previous prophylactic treatment strategies with cannabinoids in a traumatic nerve injury model demonstrated that pre-emptive cannabinoids produced greater antinociception relative to post-injury treatment [19]. Here we investigate the therapeutic efficacy of prophylactically administered WIN55,212-2, a mixed cannabinoid (CB_1_/CB_2_) agonist, and AM1710, a CB_2_-preferring agonist, on the development of chemotherapy-induced neuropathy in the paclitaxel model. Osmotic mini pumps were used to continuously infuse cannabinoids before, during, and after paclitaxel treatment, to emulate a prophylactic analgesic strategy achievable in clinical oncology settings. We compared development of mechanical and cold allodynia, both common clinical manifestations of paclitaxel-induced neuropathy [10,20]. We hypothesized that chronic prophylactic cannabinoid infusion would produce sustained suppression of paclitaxel-induced behavioral sensitization to mechanical and cold stimulation. Furthermore, we evaluated whether long-term transcriptional changes in mRNA markers of astrocytes (GFAP), microglia (CD11b), and cannabinoid receptors (CB_1_, CB_2_) would accompany long lasting anti-allodynic efficacy of cannabinoids.

## Results

### General results

**Figure 1:**
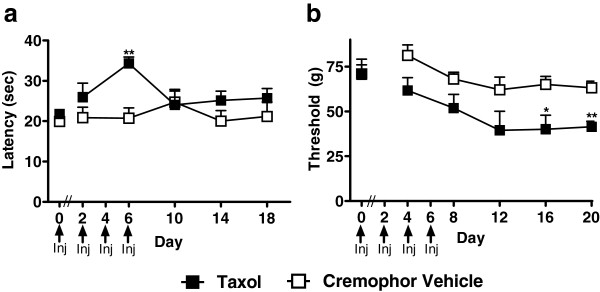
**Paclitaxel induces mechanical allodynia without producing hypersensitivity to heat. a**. Paclitaxel treatment produced transient heat hypoalgesia but no long-term changes in paw withdrawal latencies to heat whereas **b**. the same animals developed mechanical allodynia. Inj indicates days when injections of paclitaxel or cremophor vehicle occurred. Taxol; paclitaxel. *P < 0.05, **P <0.01 vs. Cremophor Vehicle, (ANOVA). N = 6 per group.

Paclitaxel-treated animals showed reduced sensitivity to heat on day 6 (F_1,10_ = 20.745, P < 0.01; Figure 1a), but not at subsequent time points (*P* > 0.16), while the same animals developed hypersensitivity to mechanical stimulation (i.e., mechanical allodynia) (F_1,10_ = 6.191, P < 0.05; Figure 1b). Based upon these results, animals implanted with osmotic pumps were evaluated for responsiveness to mechanical and cold stimulation only.

Osmotic mini pump dispersion volume was calculated by subtracting the fill volume from the residual volume in the pump reservoir following pump removal (day 22). The pump dispersion volume differed between groups in which drugs were dissolved in the DMSO:PEG400 vehicle (F_19,180_ = 2.213, *P* < 0.01). Post-hoc analysis revealed that pump dispersion volume for the Taxol-WIN55,212-2 (1 mg/kg/day s.c.) group was less than half (< 43%) of other groups dissolved in the same vehicle. No other differences were found. Mechanical withdrawal thresholds did not differ between either the right or left paw on any given day for animals tested up to 20 (*P* > 0.98) or 50 (*P* > 0.71) days post-chemotherapy treatment; therefore, withdrawal thresholds are presented as the mean of duplicate measurements, averaged across paws. Two dependent measures for cold allodynia were evaluated: percentage of paw withdrawals and duration of paw withdrawal. Duration of paw withdrawal in response to topical acetone application is a reported measure of cold allodynia [21-23]. However, we found this measure highly variable in rat subjects (data not shown) and consequently only the percentage of paw withdrawals is reported here. Percentage of paw withdrawals to cold stimulation did not differ between either paw on any given day for animals tested up to 21 (*P* > 0.33) or 51 (*P* > 0.82) days post-paclitaxel; therefore, the percentage of paw withdrawals is presented as the mean of duplicate measurements averaged across paws.

To control for any possible effects associated with the vehicle used to dissolve cannabinoids (DMSO:PEG 400 in a 1:1 ratio), a subset of animals treated with either paclitaxel or cremophor received saline in their osmotic mini pumps. No differences were detected between paclitaxel-treated animals that received vehicle (DMSO:PEG 400; n = 14) or saline (n = 4) in any behavioral parameter assessed (i.e., mechanical threshold, cold withdrawal frequency, and locomotor activity). Similarly, no differences were noted between cremophor-treated animals receiving chronic infusions of vehicle (DMSO:PEG 400; n = 8) or saline (n = 4). Therefore, vehicle and saline groups were combined for each condition and are referred to as the Taxol-vehicle group and cremophor-vehicle group, respectively.

### Body weight

**Figure 2:**
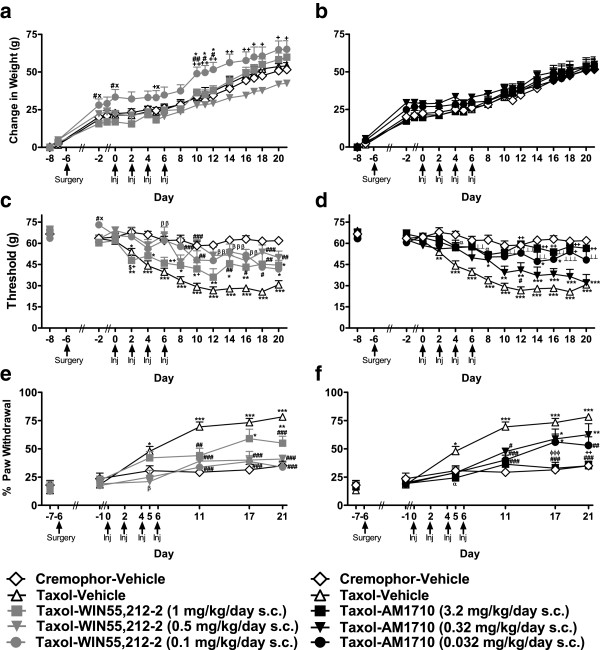
**The mixed CB**_**1**_**/CB**_**2 **_**agonist WIN55,212-2 and the CB**_**2**_**-preferring agonist AM1710 suppressed development of paclitaxel-induced mechanical and cold allodynia without significantly altering body weight. a**. WIN55,212-2 (0.1 mg/kg/day s.c.) increased whereas **b**. AM1710 did not alter body weight in paclitaxel-treated animals. Mechanical and cold allodynia were suppressed by WIN55,212-2 (0.1 and 0.5 mg/kg/day s.c.; **c**. **and e**., respectively) and AM1710 (0.032 and 3.2 mg/kg/day s.c.; **d**. **and f**., respectively). *P < 0.05, **P <0.01, ***P <0.001 vs. Cremophor-Vehicle, ^#^*P* < 0.05, ^##^*P* < 0.01, ^###^*P* <0.001 vs. Taxol-Vehicle, ^x^*P* < 0.05 vs. Taxol-Agonist (high dose), ^+^*P* < 0.05, ^++^*P* < 0.01 vs. Taxol-Agonist (middle dose), ^$^*P* < 0.05, vs. Taxol-Agonist (low dose), ^β^*P* < 0.05, ^ββ^*P* < 0.01, ^βββ^*P* < 0.001 Taxol-Agonist (middle and low doses) vs. Taxol-Vehicle,^⟂⟂^*P* < 0.01, ^⟂⟂⟂^*P* < 0.001 Taxol-Agonist (high and low doses) vs. Taxol-Vehicle, ^α^*P* < 0.05 Taxol-Agonist (all doses) vs. Taxol-Vehicle, ^ϕϕϕ^*P* <0.001 vs. Taxol-Agonist (middle and low doses). The first drug listed indicates assignment to cremophor or paclitaxel (Taxol) treatment. The second drug indicates drug administered via osmotic mini pump chronic infusion. Day numbers reference days post-chemotherapeutic treatment (i.e., negative days indicate days *prior to* chemotherapeutic treatment). Surgery indicates the day (day -6) on which osmotic mini pumps were implanted subcutaneously. (ANOVA; Dunnett and Tukey post-hoc tests). N = 8–18 per group.

Body weight did not differ between paclitaxel- or cremophor-treated animals receiving infusions of vehicle (*P* = 0.69; Figure 2a). Moreover, no differences in body weight were observed between paclitaxel-treated animals receiving either vehicle or saline (data not shown). However, cremophor-treated animals receiving saline infusions exhibited greater weight gain on days 14–21 (F_12,204_ = 8.455, *P* < 0.001, P < 0.05 for each day) relative to those receiving vehicle.

**Figure 3:**
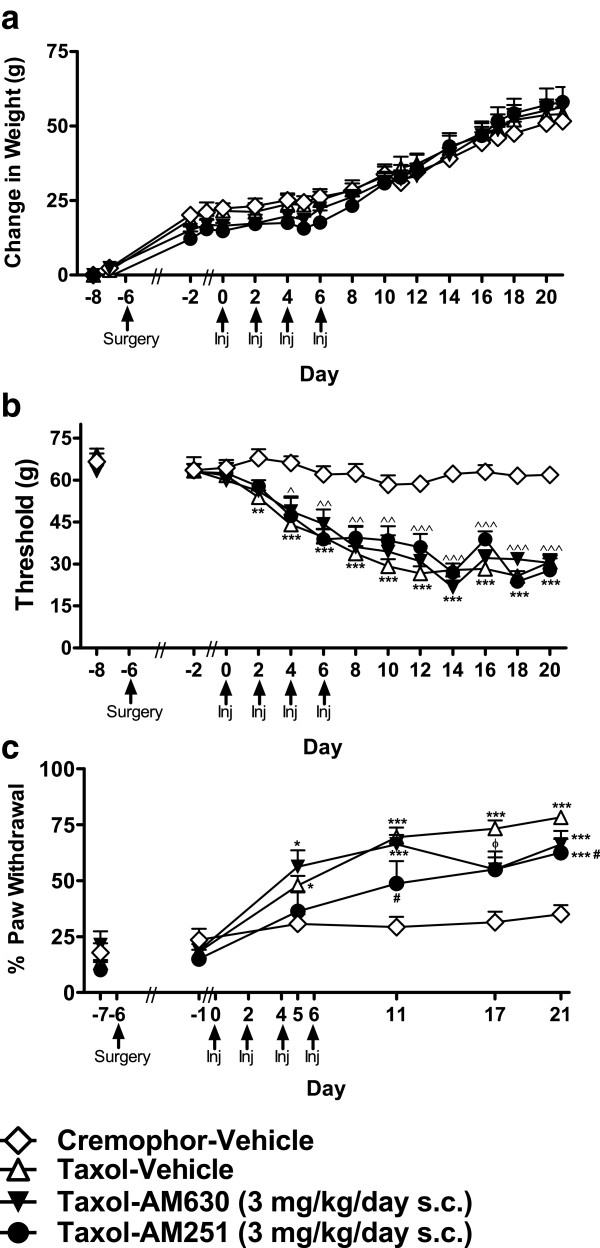
**Neither the CB**_**1 **_**antagonist AM251 nor the CB**_**2 **_**antagonist AM630 altered paclitaxel-induced mechanical or cold allodynia. a**. No changes in body weight, responsiveness to **b**. mechanical, or **c**. cold stimulation were observed in paclitaxel-treated animals receiving AM630 (3 mg/kg/day s.c.) or AM251 (3 mg/kg/day s.c.) relative to Taxol-vehicle animals. *P < 0.05, **P < 0.01, ***P <0.001 vs. Cremophor-Vehicle, ^^^*P* < 0.05, ^^^^*P* < 0.01, ^^^^^*P* < 0.001 Taxol-AM630 (3 mg/kg/day s.c.) and Taxol-AM251 (3 mg/kg/day s.c.) vs. Cremophor-Vehicle, ^#^*P* < 0.05 vs. Taxol-Vehicle, ^ϕ^*P* < 0.05 Taxol-AM630 (3 mg/kg/day s.c.) and Taxol-AM251 (3 mg/kg/day s.c.) vs. Taxol-Vehicle and Cremophor-Vehicle. Doses are in mg/kg/day s.c. (ANOVA; Dunnett and Tukey post-hoc tests). N = 10–18 per group.

Paclitaxel-treated animals receiving infusions of WIN55,212-2 (0.1 mg/kg/day s.c.) showed greater weight gain over the study (F_68,935_ = 3.932, P < 0.001; *P* < 0.05 for each comparison) relative to other groups (F_4,55_ = 2.627, *P* < 0.05; Figure 2a). Body weight did not differ in paclitaxel-treated animals receiving AM1710 (3.2, 0.32, and 0.032 mg/kg/day s.c.) (*P* > 0.86; Figure 2b) or either antagonist (*P* > 0.93; Figure 3a). Neither of the agonists altered weight gain relative to vehicle in cremophor-treated groups (*P* = 0.137; data not shown).

### Effects of prophylactic WIN55,212-2 and AM1710 treatment on paclitaxel-evoked mechanical allodynia

#### Anti-allodynic effects of the mixed CB_1_/CB_2_ agonist WIN55,212-2

Paclitaxel-treated animals receiving vehicle infusions developed mechanical allodynia relative to cremophor-treated counterparts; mechanical allodynia was apparent on day 2 and persisted until the final test day prior to pump removal (day 20) (F_48,660_ = 3.880, *P* < 0.001; P < 0.01 for each comparison; Figure 2c). WIN55,212-2 (0.1 mg/kg/day s.c.) produced a transient antinociceptive effect *prior to* paclitaxel treatment on day -2 (*P* < 0.05); this antinociceptive effect was observed relative to paclitaxel-treated groups receiving either vehicle or WIN55,212-2 (1.0 mg/kg/day s.c.). WIN55,212-2 (0.5 mg/kg/day s.c.) blocked development of paclitaxel-induced mechanical allodynia (F_4,55_ = 32.964, *P* < 0.001; Figure 2c) and normalized mechanical thresholds relative to the Taxol-vehicle group at all time points (*P* < 0.05 for each comparison). WIN55,212-2 (0.1 mg/kg/day s.c.) also suppressed the development of paclitaxel-evoked mechanical allodynia over the time course corresponding to drug delivery (*P* < 0.05 for each comparison) but failed to normalize thresholds relative to cremophor-vehicle levels.

#### Anti-allodynic effects of the CB_2_ agonist AM1710

AM1710 (3.2 and 0.032 mg/kg/day s.c.) blocked development of paclitaxel-evoked mechanical allodynia (F_4,59_ = 41.988, *P* < 0.001; Figure 2d) over the time course corresponding to drug delivery (F_48,708_ = 5.186, *P* < 0.001; *P* < 0.01 for each comparison). AM1710 (3.2 and 0.032 mg/kg/day s.c.) increased mechanical withdrawal thresholds relative to the Taxol-vehicle group beginning on day 4 and this effect was maintained for the duration of the study (*P* < 0.05 for each comparison). The high dose of AM1710 (3.2 mg/kg/day s.c.) preferentially increased mechanical paw withdrawal thresholds relative to the middle dose (0.32 mg/kg/day s.c.) from days 12–20 (*P* < 0.05 for each comparison). Moreover, AM1710 (3.2 mg/kg/day s.c) normalized paw withdrawal thresholds in paclitaxel-treated animals to those observed in the cremophor-vehicle group at all time points.

### Effects of prophylactic WIN55,212-2 and AM1710 treatment on paclitaxel-evoked cold allodynia

#### Anti-allodynic effects of the mixed CB_1_/CB_2_ agonist WIN55,212-2

Paclitaxel-induced cold allodynia developed by day 5 and was stable until the final test day associated with drug delivery (day 21) (F_20,275_ = 7.197, *P* < 0.001; *P* < 0.05 for each comparison; Figure 2e). The middle and low doses of WIN55,212-2 (0.5 and 0.1 mg/kg/day s.c.) blocked development of cold allodynia in paclitaxel-treated animals (F_4,55_ = 11.428, *P* < 0.001, *P* < 0.05 for each comparison; Figure 2e) for the duration of drug delivery. The high dose of WIN55,212-2 (1 mg/kg/day s.c.) failed to fully suppress development of paclitaxel-induced cold allodynia. However, animals in this group nonetheless showed protection against cold allodynia relative to paclitaxel-vehicle treated animals at some observation intervals (i.e., days 11 and 21; *P* < 0.001).

#### Anti-allodynic effects of the CB_2_-preferring agonist AM1710

AM1710 (3.2 mg/kg/day s.c.) suppressed development of paclitaxel-induced cold allodynia (F_4,59_ = 14.299, *P* < 0.001; *P* < 0.05 for each comparison) over the time course of drug delivery (F_20,295_ = 6.871, *P* < 0.001; *P* < 0.05 for each comparison; Figure 2f). Lower doses of AM1710 (0.32 and 0.032 mg/kg/day s.c.) had a shorter duration of action; suppression of cold allodynia was only observed until day 11 (*P* < 0.05 for each comparison).

### Comparison of anti-allodynic efficacy of AM1710 and WIN55,212-2

**Figure 4:**
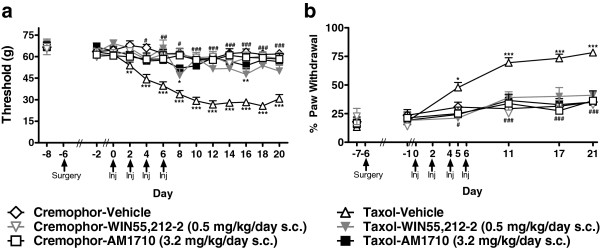
**The mixed cannabinoid CB**_**1**_**/CB**_**2 **_**agonist WIN55,212-2 (0.5 mg/kg/day s.c.) and the CB**_**2**_**-preferring agonist, AM1710 (3.2 mg/kg/day s.c.) suppressed the development of both a. mechanical and b. cold allodynia associated with paclitaxel treatment.** No antinociception was observed in cremophor animals treated with either cannabinoid agonist in response to mechanical or cold stimulation.*P <0.05, **P <0.01,***P <0.001 vs. Cremophor-Vehicle, ^#^*P* < 0.05, ^##^*P* <0.01, ^###^*P* <0.001 All conditions vs. Taxol-Vehicle (ANOVA; Dunnett and Tukey post-hoc tests). N = 10–18 per group.

We compared the anti-allodynic efficacy of the maximally efficacious doses of WIN55,212-2 (0.5 mg/kg/day s.c.) and AM1710 (3.2 mg/kg/day s.c.) under analogous conditions (Figure 4). Both WIN55,212-2 (0.5 mg/kg/day s.c.) and AM1710 (3.2 mg/kg/day s.c.) elevated mechanical withdrawal thresholds in paclitaxel-treated relative to cremophor-vehicle treated rats (F_5,66_ = 66.292, *P* < 0.001; *P* < 0.01 for each comparison; Figure 4a) from day 4 through the final test day corresponding to drug delivery (F_60,792_ = 4.888, *P* < 0.001; *P* < 0.05 for each comparison). WIN55,212-2 (0.5 mg/kg/day s.c.) normalized mechanical withdrawal thresholds in paclitaxel-treated groups with two exceptions; a transient drop in threshold on days 8 and 16 was observed relative to the cremophor-vehicle group (*P* < 0.05 for each comparison). By contrast, AM1710 (3.2 mg/kg/day s.c.) effectively normalized mechanical thresholds in paclitaxel-treated animals to those observed in the cremophor-vehicle group. WIN55,212-2 and AM1710 suppressed development of paclitaxel-induced cold allodynia with similar efficacy (F_5,66_ = 12.365, *P* < 0.001; *P* < 0.05 for each comparison; Figure 4b) over the time course (F_25,330_ = 6.892, *P* < 0.001). Neither agonist produced antinociception to either mechanical or cold stimulation in animals that received cremophor vehicle in lieu of paclitaxel.

### Pharmacological specificity

#### Mechanical allodynia

##### Pharmacological specificity of WIN55,212-2-mediated anti-allodynia

**Figure 5:**
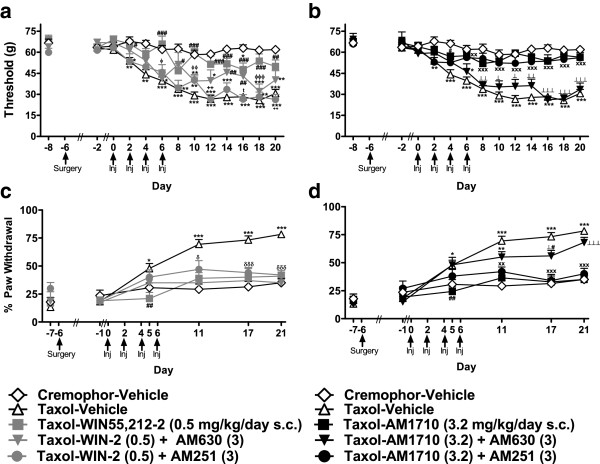
**Pharmacological specificity of cannabinoid agonist-induced suppression of paclitaxel-induced mechanical and cold allodynia. a**. WIN55,212-2 (0.5 mg/kg/day s.c.)-mediated suppression of paclitaxel-induced mechanical allodynia was dominated by CB_1_ receptor activation with some involvement of CB_2_ receptors. **b**. The AM1710 (3.2 mg/kg/day s.c.)-induced suppression of paclitaxel-induced mechanical allodynia was blocked by AM630 (3 mg/kg/day s.c.) but not AM251 (3 mg/kg/day s.c.)). **c**. Neither AM630 (3 mg/kg/day s.c.) nor AM251 (3 mg/kg/day s.c.) reliably altered the anti-allodynic effects of WIN55,212-2 (0.5 mg/kg/day s.c.) following acetone application. **d**. AM630 (3 mg/kg/day s.c.), but not AM251 (3 mg/kg/day s.c.), blocked the anti-allodynic effects of AM1710 (3.2 mg/kg/day s.c.) to cold stimulation. *P < 0.05, **P < 0.01, ***P < 0.001 vs. Cremophor-Vehicle, ^#^*P* < 0.05, ^##^*P* < 0.01, ^###^*P* < 0.001 vs. Taxol-Vehicle, ^++^*P* < 0.01 vs. Taxol-Agonist, ^xx^*P* < 0.01, ^xxx^*P* < 0.001 Taxol-Agonist and Taxol-Agonist + AM251 (3) vs. Taxol-Vehicle, ^t^*P* < 0.05 vs. Taxol-Agonist, Taxol-Agonist + AM630 (3), and Cremophor-Vehicle, ^ϕ^*P* < 0.05, ^ϕϕ^*P* < 0.01, ^ϕϕϕ^*P* < 0.001 Taxol-Agonist + AM630 (3) and Taxol-Agonist + AM251 (3) vs. Taxol-Agonist, ^⟂^*P* < 0.05, ^⟂⟂^*P* < 0.01, ^⟂⟂⟂^*P* < 0.001 vs. Taxol-Agonist, Taxol-Agonist + AM251 (3), and Cremophor-Vehicle, ^δ^*P* < 0.05, ^δδδ^*P* <0.001 Taxol-Agonist, Taxol-Agonist + AM251 (3), and Taxol-Agonist + AM630 (3) vs. Taxol-Vehicle. Doses are in mg/kg/day s.c. (ANOVA; Dunnett and Tukey post-hoc tests). N = 10–18 per group.

Simultaneous infusion of AM251 (3 mg/kg/day s.c.) suppressed anti-allodynic effects of WIN55,212-2 (0.5 mg/kg/day s.c.) (F_4,57_ = 38.335, *P* < 0.001; Figure 5a) beginning on day 6 and lasting through the final test day (day 20) corresponding to active drug delivery (F_48, 684_ = 4.112, *P* < 0.001; *P* < 0.05 for each comparison). The CB_2_-specific antagonist AM630 (3 mg/kg/day) showed inconsistent efficacy in blocking anti-allodynic effects of WIN55,212-2 (0.5 mg/kg/day s.c.) (*P* < 0.05 for each comparison).

##### Pharmacological specificity of AM1710-mediated anti-allodynia

Simultaneous infusion of AM630 (3 mg/kg/day s.c.) suppressed anti-allodynic effects of AM1710 (3.2 mg/kg/day s.c.) (F_4,61_ = 44.885, *P* < 0.001, Figure 5b) in paclitaxel-treated rats from days 8 through 20 (F_48, 732_ = 6.161, *P* < 0.001, *P* < 0.05 for each comparison). By contrast, AM251 (3 mg/kg/day s.c.) failed to block the anti-allodynic effects of AM1710 (3.2 mg/kg/day s.c.); thresholds differed reliably from paclitaxel-vehicle treatment throughout the observation interval (*P* < 0.05 for each comparison).

##### Effects of antagonists administered alone

Neither AM630 (3 mg/kg/day s.c.) nor AM251 (3 mg/kg/day) altered paclitaxel-induced mechanical allodynia relative to vehicle treatment. Paclitaxel-induced mechanical allodynia developed equivalently in groups receiving infusions of either AM630 (3 mg/kg/day s.c.) or AM251 (3 mg/kg/day) relative to cremophor-vehicle (F_3,44_ = 58.077, *P* < 0.001, *P* < 0.05 for each comparison; Figure 3b) throughout the time course (F_36,528_ = 6.134, *P* < 0.001).

#### Cold allodynia

##### Pharmacological specificity of WIN55,212-2 effects on cold allodynia

WIN55,212-2 (0.5 mg/kg/day s.c.)-induced suppression of cold allodynia was not reliably blocked by either AM630 (3 mg/kg/day s.c.) or AM251 (3 mg/kg/day s.c.) (F_4,57_ = 10.343, *P* < 0.001; Figure 5c) (F_20,285_ = 8.415, *P* < 0.001, *P* < 0.05 for each comparison).

##### Pharmacological specificity of AM1710-mediated anti-allodynia

The AM1710 (3.2 mg/kg/day s.c.)-induced suppression of cold allodynia was blocked by AM630 (3 mg/kg/day s.c.) (F_4,61_ = 14.178, *P* < 0.001, Figure 5d) but not AM251 (3 mg/kg/day s.c.). This blockade was fully apparent by days 17 and 21 post-paclitaxel (F_20,305_ = 8.201, *P* < 0.001; *P* < 0.05 for each comparison). Cold allodynia developed similarly in paclitaxel-treated rats that received AM1710 (3.2 mg/kg/day s.c.) together with AM251 (3 mg/kg/day s.c.) and AM1710 (3.2 mg/kg/day s.c.) alone.

##### Effects of antagonists administered alone

Paclitaxel-treated animals receiving either AM630 (3 mg/kg/day s.c.) or AM251 (3 mg/kg/day s.c.) developed cold allodynia (F_3,44_ = 12.138, *P* < 0.001; Figure 3c) relative to cremophor-vehicle control animals (F_15,220_ = 7.742, *P* < 0.001, *P* < 0.05 for each comparison). Taxol-AM251 (3 mg/kg/day s.c.) animals showed attenuated cold allodynia relative to Taxol-vehicle animals on days 11, 17 and 21 (*P* < 0.05 for each comparison) Responsiveness to acetone was, nonetheless, elevated relative to cremophor-vehicle treatment at each time point (*P* < 0.05 for each comparison).

### Protective effects of WIN55,212-2 and AM1710 following drug removal

#### Mechanical allodynia

**Figure 6:**
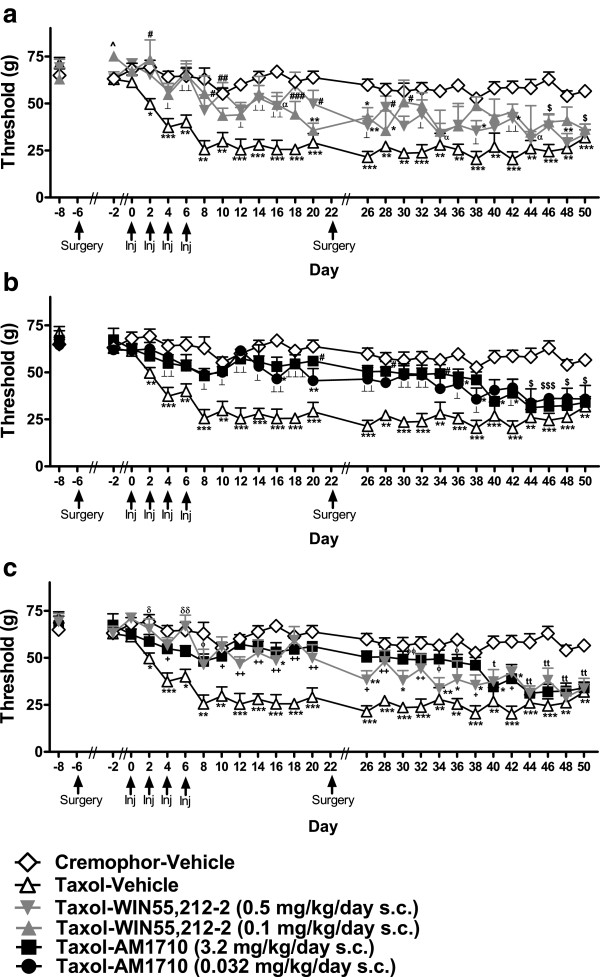
**Protective effects of WIN55,212-2 and AM1710 on paclitaxel-induced mechanical allodynia following drug removal. a**. WIN55,212-2 (0.5 mg/kg/day s.c.) suppressed paclitaxel-induced mechanical allodynia 11 days (day 32) following drug removal. **b**. AM1710 (3.2 and 0.032 mg/kg/day s.c.) suppressed hypersensitivity to mechanical stimulation up to 17 days following drug removal (until day 38). **c**. AM1710 (3.2 mg/kg/day s.c.) produced a longer duration of protection against paclitaxel-induced mechanical allodynia relative to WIN55,212-2 (0.5 mg/kg/day s.c.). *P < 0.05, **P < 0.01, ***P <0.001 vs. Cremophor-Vehicle, ^#^*P* < 0.05, ^##^*P* <0.01, ^###^*P* <0.001 vs. Taxol-Vehicle, ^⟂^*P* < 0.05, ^⟂⟂^*P* < 0.01, ^⟂⟂⟂^*P* < 0.001 Taxol-Agonist (both doses) vs. Taxol-Vehicle, ^*P* < 0.05 vs. all groups, ^$^*P* < 0.05, ^$$$^*P* < 0.001 Taxol-Agonist (both doses) vs. Cremophor-Vehicle, ^α^*P* < 0.05 Taxol-WIN55,212-2 (0.5 mg/kg/day s.c.) vs. Cremophor-Vehicle, ^+^*P* < 0.05, ^++^*P* < 0.01, Taxol-AM1710 (3.2 mg/kg/day s.c.) and Taxol-WIN55,212-2 (0.5 mg/kg/day s.c.) vs. Taxol-Vehicle, ^ϕ^*P* < 0.05, ^ϕϕ^*P* <0.01 Taxol-AM1710 (3.2 mg/kg/day s.c.) vs. Taxol-Vehicle, ^δ^*P* < 0.05, ^δδ^*P* < 0.01 Taxol-WIN55,212-2 (0.5 mg/kg/day s.c.) vs. Taxol-Vehicle, ^t^*P* < 0.05, ^tt^*P* < 0.01 Taxol-AM1710 (3.2 mg/kg/day s.c.) and Taxol-WIN55,212-2 (0.5 mg/kg/day s.c.) vs. Cremophor-Vehicle (ANOVA; Dunnett and Tukey post hoc tests). N = 4–8 per group.

Paclitaxel produced long-lasting mechanical allodynia in rats receiving infusions of vehicle relative to cremophor-vehicle treatment (F_75,500_ = 2.218, *P* < 0.01, *P* < 0.05 for each comparison; Figure 6a); these effects persisted until the final test day (day 50). We next examined the protective effects of WIN55,212-2 and AM1710 following cessation of drug delivery (Figure 6). WIN55,212-2 (0.5 and 0.1 mg/kg/day s.c., delivered from days -6 through 22) blocked the development of paclitaxel-induced mechanical allodynia (F_3,20_ = 48.189, *P* < 0.001; Figure 6a) for approximately 11 days following cessation of drug delivery (F_75,500_ = 2.218, *P* < 0.01, *P* < 0.05 for each comparison). Similarly, AM1710 (3.2 mg/kg/day s.c.) protected against development of paclitaxel-induced mechanical allodynia for 17 days following drug removal (i.e., day 38); (F_3,22_ = 41.754, *P* < 0.001, *P* < 0.05 for each comparison; Figure 6b). The low dose of AM1710 (0.032 mg/kg/day s.c.) also increased paw withdrawal thresholds up to 17 days following drug removal (*P* < 0.01 for each comparison); however, thresholds in this group failed to differ from the paclitaxel-vehicle condition on several days (days 28 and 34), suggesting that mechanical allodynia was beginning to develop. The high dose of AM1710 (3.2 mg/kg/day s.c.) produced longer protection (F_75,550_ = 2.584, P < 0.001, *P* < 0.05 for each comparison; Figure 6c) against mechanical allodynia development compared to WIN55,212-2 (F_3,22_ = 69.008, P < 0.001).

#### Cold allodynia

**Figure 7:**
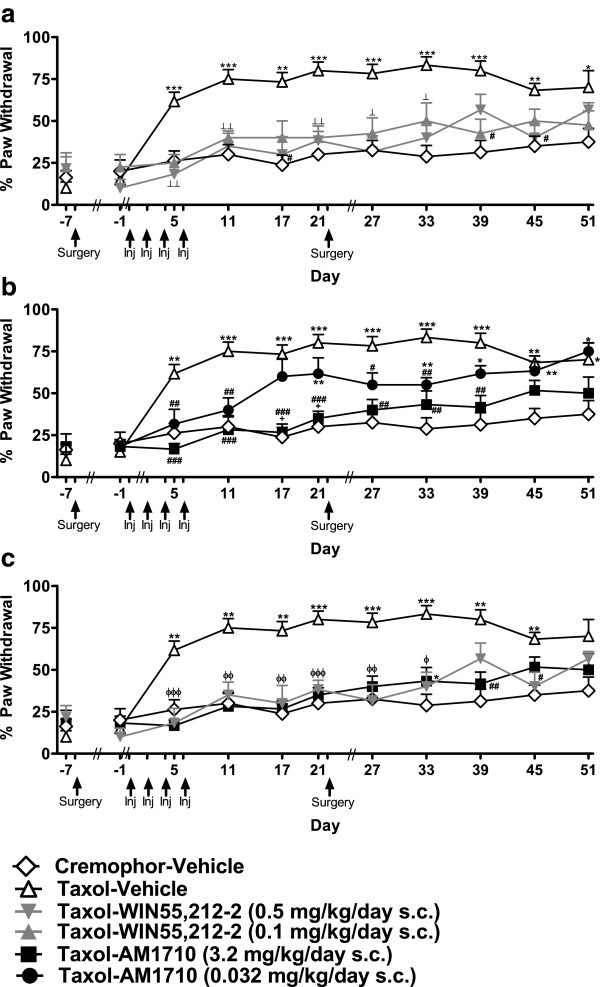
**Protective effects of WIN55,212-2 and AM1710 on paclitaxel-induced cold allodynia following drug removal. a**. WIN55,212-2 (0.5 and 0.1 mg/kg/day s.c.) suppressed paclitaxel-induced cold allodynia for 12 and 18 days (until day 33 and 39, respectively) following cessation of drug delivery. **b**. AM1710 (3.2 mg/kg/day s.c.) suppressed cold allodynia for 18 days following drug removal (until day 39). **c**. AM1710 (3.2 mg/kg/day s.c.) produced a longer duration of protection against cold allodynia compared to WIN55,212-2 (0.5 mg/kg/day s.c.). *P < 0.05, **P < 0.01, ***P <0.001 vs. Cremophor-Vehicle, ^#^*P* < 0.05, ^##^*P* <0.01, ^###^*P* <0.001 vs. Taxol-Vehicle, ^⟂^*P* < 0.05, ^⟂⟂^*P* < 0.01 Taxol-Agonist (both doses) vs. Taxol-Vehicle, ^ϕ^*P* < 0.05, ^ϕϕ^*P* <0.01, ^ϕϕϕ^*P* <0.001 Taxol-AM1710 (3.2 mg/kg/day s.c.) and Taxol-WIN-55,212-2 (0.5 mg/kg/day s.c.) vs. Taxol-Vehicle (ANOVA; Dunnett and Tukey post-hoc tests). N = 4–8 per group.

Paclitaxel increased responsiveness to acetone in animals receiving infusions of vehicle throughout the time course (F_30,200_ = 3.784, *P* < 0.001; *P* < 0.05 for each comparison; Figure 7a). WIN55,212-2 (0.5 and 0.1 mg/kg/day s.c.) suppressed development of paclitaxel-induced cold allodynia (F_3,20_ = 12.367, *P* < 0.001; Figure 7a) up to 12 (day 33) and 18 days (day 39) following cessation of analgesic drug delivery, respectively. AM1710 (3.2 mg/kg/day s.c.) suppressed development and postponed emergence of cold allodynia (F_3,22_ = 16.132, *P* < 0.001; *P* < 0.05 for each comparison; Figure 7b) for 18 days following cessation of drug delivery (F_30,220_ = 4.709, *P* < 0.001; *P* < 0.05 for each comparison). The low dose of AM1710 (0.032 mg/kg/day s.c.) suppressed cold allodynia through day 33 (*P* < 0.05 for each comparisons), indicating a shorter duration of protection relative to the high dose. Both AM1710 (3.2 mg/kg/day s.c.) and WIN55,212-2 (0.5 mg/kg/day s.c.) protected against development of paclitaxel-induced cold allodynia (F_3,22_ = 13.216, P < 0.001, *P* < 0.05 for each comparison; Figure 7c) over the time course (F_30,220_ = 4.439, P < 0.001) The high dose of AM1710 (3.2 mg/kg/day s.c.) delayed the emergence of paclitaxel-induced cold allodynia longer than WIN55,212-2 (0.5 mg/kg/day s.c.).

#### Pharmacological specificity of protective effects

##### Mechanical allodynia

**Figure 8:**
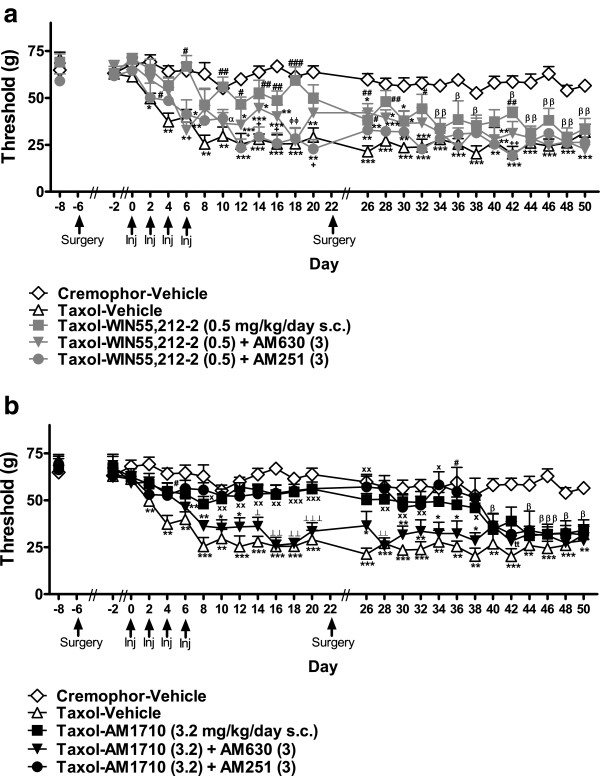
**Pharmacological specificity of cannabinoid-mediated protection against paclitaxel-induced mechanical allodynia following drug removal. a**. WIN55,212-2 (0.5 mg/kg/day s.c.)-mediated anti-allodynia following drug removal was dominated by CB_1_ receptor activation. **b**. The protective effects of AM1710 were blocked by AM630 (3 mg/kg/day s.c.) but not AM251 (3 mg/kg/day s.c.)). *P < 0.05, **P < 0.01, ***P < 0.001 vs. Cremophor-Vehicle, ^#^*P* < 0.05, ^##^*P* < 0.01, ^###^*P* < 0.001 vs. Taxol-Vehicle, ^x^*P* < 0.05, ^xx^*P* < 0.01, ^xxx^*P* < 0.001 Taxol-Agonist and Taxol-Agonist + AM251 (3) vs. Taxol-Vehicle, ^⟂^*P* < 0.05, ^⟂⟂^*P* < 0.01, ^⟂⟂⟂^*P* < 0.001 vs. Taxol-Agonist, Taxol-Agonist + AM251 (3) and Cremophor-Vehicle, ^α^*P* < 0.05 Taxol-Agonist + AM630 (3) vs. Cremophor-Vehicle, ^β^*P* < 0.05, ^ββ^*P* < 0.01, ^βββ^*P* < 0.001 Taxol-Agonist, Taxol-Agonist + AM251 (3), and Taxol-Agonist + AM630 (3) vs. Cremophor-Vehicle, ^ϕϕ^*P* < 0.01 Taxol-Agonist + AM251 (3), and Taxol-Agonist + AM630 (3) vs. Taxol-Agonist and Cremophor-Vehicle, ^+^*P* < 0.05, ^++^*P* < 0.01 vs. Taxol-Agonist, ^tt^*P* < 0.01 Taxol-Agonist + AM251 (3) and Taxol-Agonist + AM630 (3) vs. Cremophor-Vehicle. Doses are in mg/kg/day s.c. (ANOVA; Dunnett and Tukey post-hoc tests). N = 6–8 per group.

Animals in the Taxol-WIN55,212-2 (0.5) + AM630 (3) group did not fully develop mechanical allodynia until day 34 (F_4,27_ = 41.884, *P* < 0.001, *P* < 0.05 for each comparison; Figure 8a), consistent with the anti-allodynic efficacy of WIN55,212-2 (0.5 mg/kg/day s.c.) alone. The AM1710 (3.2 mg/kg/day s.c.) + AM251 (3 mg/kg/day s.c.) group only developed mechanical allodynia after day 38 (F_4,27_ = 25.046, *P* < 0.001; *P* < 0.05 for each comparison; Figure 8b), when protective effects of AM1710 (3.2 mg/kg/day s.c.) alone were no longer apparent.

##### Cold allodynia

**Figure 9:**
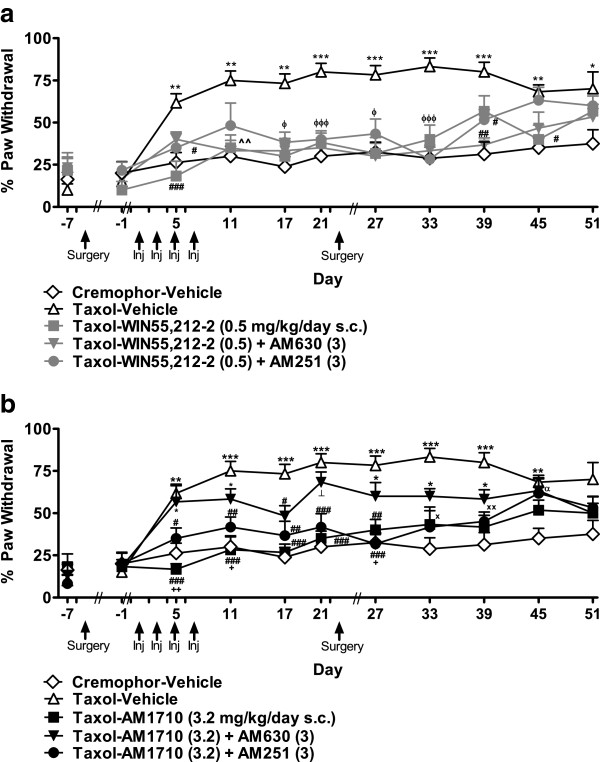
**Pharmacological specificity of cannabinoid-mediated protection against paclitaxel-induced cold allodynia following drug removal. a**. WIN55,212-2 (0.5 mg/kg/day s.c.)-induced protection against paclitaxel-induced cold allodynia was not blocked by either a CB_1_ (AM251, 3 mg/kg/day s.c.) or CB_2_ (AM630, 3 mg/kg/day s.c.) antagonist. **b**. Protective anti-allodynic effects associated with AM1710 (3.2 mg/kg/day s.c.) were mediated via CB_2_ receptor activation. *P < 0.05, **P < 0.01, ***P < 0.001 vs. Cremophor-Vehicle, ^#^*P* < 0.05, ^##^*P* < 0.01, ^###^*P* < 0.001 vs. Taxol-Vehicle, ^+^*P* < 0.05, ^++^*P* < 0.01 vs. Taxol-Agonist + AM630 (3), ^x^*P* < 0.05**, **^xx^*P* < 0.01, Taxol-Agonist and Taxol-Agonist + AM251 (3) vs. Taxol-Vehicle, ^⟂^*P* < 0.05 vs. Taxol-Agonist, Taxol-Agonist + AM251 (3) and Cremophor-Vehicle ^ϕ^*P* < 0.05, ^ϕϕϕ^*P* < 0.001 Taxol-Agonist, Taxol-Agonist + AM251 (3) and Taxol-Agonist + AM630 (3) vs. Cremophor-Vehicle, ^^*P* < 0.01 Taxol-Agonist and Taxol-Agonist + AM630 (3) vs. Taxol-Vehicle, ^α^*P* < 0.05 Taxol-Agonist + AM630 (3) and Taxol-Agonist + AM251 (3) vs. Cremophor-Vehicle. All doses are in mg/kg/day s.c. (ANOVA; Dunnett and Tukey post-hoc tests). N = 6–8 per group.

Neither AM630 (3 mg/kg/day s.c.) nor AM251 (3 mg/kg/day s.c.) blocked anti-allodynic effects of WIN55,212-2 (0.5 mg/kg/day s.c.) (F_4,27_ = 8.965, *P* < 0.001, *P* < 0.05 for each comparison; Figure 9a). Both groups showed anti-allodynic effects relative to the cremophor-vehicle group for 18 days following drug removal (i.e., up to day 39) (F_40,270_ = 3.677, *P* < 0.001; *P* < 0.05 for each comparison). The anti-allodynic effects observed in the WIN55,212-2 blockade conditions outlasted protective effects observed with WIN55,212-2 (0.5 mg/kg/day s.c.) administered alone. AM630 (3 mg/kg/day s.c.) blocked anti-allodynic effects of AM1710 (3.2 mg/kg/day s.c.) in paclitaxel-treated animals (F_4,27_ = 12.388, *P* < 0.001, *P* < 0.05 for each comparison; Figure 9b) until day 39 (F_40,270_ = 3.687, *P* < 0.001). By contrast, the Taxol-AM1710 (3.2 mg/kg/day s.c.) + AM251 (3 mg/kg/day s.c.) group did not develop cold allodynia until day 45 (*P* < 0.05 for each comparison).

### Locomotor activity

Total distance traveled did not differ in paclitaxel- or cremophor-vehicle groups either during (day 19: *P* > 0.11) or after (day 31: *P* > 0.19) chronic drug infusion (Table 1). Moreover, antagonists did not alter locomotor activity relative to vehicle during infusion (day 19: *P* > 0.31). The combination of WIN55,212-2 (0.5 mg/kg/day s.c.) with AM630 (3 mg/kg/day s.c.) increased total distance traveled (day 19: F_5,54_ = 2.951, *P* < 0.05; *P* < 0.05 for relevant comparison; Table 1) in paclitaxel-treated animals relative to cremophor-vehicle animals. After completion of chronic infusions, paclitaxel-treated animals that previously received WIN55,212-2 (0.5 mg/kg/day s.c.) in combination with AM630 (3 mg/kg/day s.c.) also showed increased distance traveled relative to WIN55,212-2 (0.5 mg/kg/day s.c.) alone (day 31: F_5,30_ = 2.769, *P* < 0.05; *P* < 0.05 for relevant comparison; Table 1). There were no differences in distance traveled in any AM1710-treated group at any time point (day 19: *P* > 0.13; day 31: *P* > 0.19; Table 1).

**Table 1:** Locomotor activity during (Day 19) and after (Day 31) chronic drug infusions

**Group**	**Distance traveled**	
	**Day 19**	**Day 31**
Taxol-Vehicle	9108.7 ± 390.5	8369.0 ± 161.7
Cremophor-Vehicle	8186.0 ± 355.6	7843.0 ± 275.2
WIN55,212-2 (0.1)	9652.2 ± 396.2	8986.9 ± 1023.7
WIN55,212-2 (0.5)	9100.4 ± 312.7	7679.7 ± 249.0
WIN-2 (0.5) + AM630 (3)	9827.7 ± 333.9*	9407.0 ± 273.4^+^
WIN-2 (0.5) + AM251 (3)	8739.3 ± 413.4	8699.2 ± 381.1
AM1710 (0.032)	9482.2 ± 419.9	8263.9 ± 423.6
AM1710 (3.2)	8637.4 ± 412.4	7976.1 ± 469.5
AM1710 (3.2) + AM630 (3)	8562.2 ± 364.2	7577.3 ± 499.3
AM1710 (3.2) + AM251 (3)	8378.1 ± 300.7	7269.8 ± 238.3
AM630 (3)	8016.6 ± 482.3	‒‒‒‒
AM251 (3)	8442.1 ± 420.1	‒‒‒‒

Data are mean ± s.e.mean (n = 4-14 per group). All groups received paclitaxel (Taxol) with the exception of the cremophor group receiving vehicle via chronic infusion. Statistical comparisons are denoted with line divisions. All divisions within the table were compared against the cremophor- and paclitaxel-vehicle control animals. WIN-2, WIN55,212-2. Doses of drugs indicated parenthetically are in mg/kg/day s.c. ^*^P < 0.05 vs Cremophor-Vehicle, ^+^*P* < 0.05 vs. Taxol-WIN55,212-2 (0.5 mg/kg/day s.c.) (ANOVA, Tukey Post Hoc).

### Lumbar spinal cord mRNA levels of GFAP, CD11b, CB_1_ and CB_2_ receptors

**Figure 10:**
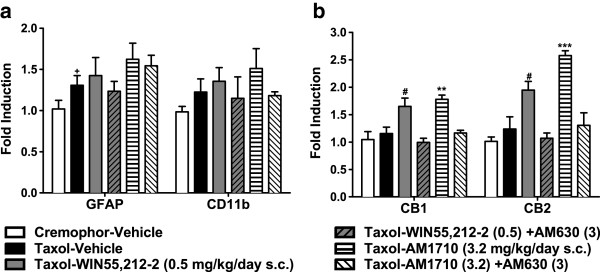
**Impact of paclitaxel and long term drug infusions on mRNA levels of astrocytes, microglia, and cannabinoid receptors in lumbar spinal cord. a**. Paclitaxel marginally altered long term expression of mRNA markers of astrocytes (GFAP) but not microglia (CD11b) in lumbar spinal cord (L4-L6) tissue samples relative to cremophor conditions. Chronic infusions of the mixed CB_1_/CB_2_ agonist WIN55,212-2 (0.5 mg/kg/day s.c.) and the CB_2_-preferring agonist AM1710 (3.2 mg/kg/day s.c.) did not alter either GFAP or CD11b **b**. but increased both CB_1_ and CB_2_ mRNA expression in the same tissues. Transcriptional regulation of CB_1_ and CB_2_ mRNAs were blocked by the CB_2_ antagonist AM630 (3 mg/kg/day s.c.). ^+^*P* = 0.059 vs. cremophor-vehicle (planned comparison one-tailed *t*-test), **P < 0.01, ***P < 0.001, vs. all groups except Taxol-WIN55,212-2 (0.5 mg/kg/day s.c.), # *P* < 0.05 vs. Taxol-Vehicle, Cremophor-Vehicle, and Taxol-WIN55,212-2 (0.5) + AM630 (3) (ANOVA; Dunnett and Tukey post hoc tests). N = 4 per group.

To understand the potential molecular targets mediating the suppression of paclitaxel-induced neuropathy by WIN55212-2 and AM1710 after cessation of drug delivery, we examined the mRNA levels of markers of astrocytes and microglia as well as CB_1_ and CB_2_ receptor mRNA levels. We used RT-PCR to measure the mRNA levels of the astrocytic marker glial fibrillary acidic protein (GFAP) and microglial marker cluster of differentiation molecule 11B (CD11b) (Figure 10a). RT-PCR analysis revealed a trend towards increased expression of GFAP (*P* = 0.059, one-tailed planned comparison *t*-test; Figure 10a) in lumbar spinal cords of paclitaxel- relative to cremophor-vehicle controls on day 22. No alterations in CD11b mRNA levels were observed at the same time point (*P* = 0.413). Neither infusion of WIN55,212-2 (0.5 mg/kg/day s.c.) nor AM1710 (3.2 mg/kg/day s.c.) altered GFAP or CD11b mRNA expression in paclitaxel-treated animals (day 22; *P* > 0.122 for each comparison; Figure 10a). Cannabinoid receptor activation by chronic agonists may produce compensatory changes in receptor levels [16], and pathological pain may alter expression levels of cannabinoid receptors [24-27]. We, therefore, measured levels of CB_1_ and CB_2_ mRNA after various treatments (Figure 10b). Both WIN55,212-2 (0.5 mg/kg/day s.c.) and AM1710 (3.2 mg/kg/day s.c.) increased mRNA expression of cannabinoid CB_1_ (F_5,23_ = 9.527, *P* < 0.001) and CB_2_ (F_5,23_ = 15.117, *P* < 0.001; Figure 10b) receptors in lumbar spinal cord. These agonist-induced increases in CB_1_ and CB_2_ receptor mRNA expression were blocked in animals that received concurrent administration of AM630 (3 mg/kg/day s.c.) (*P* < 0.05 for each comparison).

## Discussion

Prophylactic administration of cannabinoid analgesics protected against the development of paclitaxel-induced hypersensitivities to mechanical and cold stimulation in a preventative fashion. Both the mixed cannabinoid CB_1_/CB_2_ agonist WIN55,212-2 and the CB_2_ agonist AM1710 blocked development of paclitaxel-induced mechanical and cold allodynia. Strikingly, the protective prophylactic effects of both WIN55,212-2 and AM1710 were preserved following drug removal, with the CB_2_-specific agonist providing a longer duration of protection against allodynia development for both mechanical and cold modalities. In our study, paclitaxel produced marked mechanical and cold allodynia but not heat hyperalgesia, as observed in a different dosing paradigm (cumulative dose: 4 mg/kg i.p.) [28]. In vehicle (cremophor) treated controls, the most efficacious doses of these cannabinoids also failed to produce antinociception, suggesting that cannabinoids were anti-allodynic rather than analgesic under these conditions.

### Prophylactic WIN55,212-2 suppresses paclitaxel-induced mechanical and cold allodynia

WIN55,212-2 (0.5 and 0.1 mg/kg/day s.c.) suppressed the development of paclitaxel-induced mechanical and cold allodynia both during drug delivery and following drug removal. Our study is the first to evaluate duration of efficacy, dose response, and pharmacological specificity of prophylactic WIN55,212-2. Anti-allodynic effects of both doses were present 11 (mechanical) and 12 (cold) days following cessation of drug delivery. WIN55,212-2 (0.5 mg/kg/day s.c.)-induced suppression of mechanical hypersensitivity was dominated by CB_1_ receptor activation because anti-allodynic efficacy was blocked by AM251. The CB_2_ antagonist AM630 (3 mg/kg/day s.c.) prevented anti-allodynic efficacy of AM1710 but failed to eliminate WIN55,212-2-mediated anti-allodynia. Interestingly, blockade of WIN55,212-2-mediated anti-allodynic effects to cold was not achieved with either antagonist. However, the same antagonist infusion conditions blocked either WIN55,212-2 mediated suppression of mechanical allodynia (AM251) or AM1710-mediated suppression of both mechanical and cold allodynia (AM630), documenting efficacy of antagonist infusion conditions employed here.

We could find only one report of WIN55,212-2-induced suppression of cold allodynia in a neuropathic pain model (spinal nerve ligation) where pharmacological specificity was assessed; anti-allodynic effects were blocked by a CB_1_ (SR141716a) but not a CB_2_ (SR144528) antagonist [29]. Blockade of both CB_1_ and CB_2_ receptors may be required to fully prevent anti-allodynic effects of WIN55,212-2. However, limitations in compound solubility prohibited co-administration of both antagonists in one pump.

Few studies have examined cannabinoid-mediated modulation of cold allodynia and/or its development in neuropathic pain models and more work is necessary to determine functional contributions of each receptor. WIN55,212-2 (0.5 mg/kg/day s.c.) treatment increased both CB_1_ and CB_2_ receptor mRNA expression within lumbar spinal cord of paclitaxel-treated animals on day 22, an effect blocked by concurrent AM630 (3 mg/kg/day s.c.) treatment. WIN55,212-2 also ameliorates established paclitaxel-induced nociception [13] and repeated administration (1 mg/kg i.p. × 14 days) prevents nociception development during drug delivery [11].

### Prophylactic AM1710 suppresses paclitaxel-induced mechanical and cold allodynia

Strikingly, doses of AM1710 as low as 0.032 mg/kg/day blocked the development of paclitaxel-induced allodynia in our study and these effects were preserved for approximately three weeks following cessation of drug delivery. Prophylactic AM1710 treatment suppressed development of both paclitaxel-induced mechanical and cold allodynia, with high (3.2 mg/kg/day s.c.) and low (0.032 mg/kg/day s.c.) doses exhibiting the greatest efficacy. A similar U-shaped dose response curve was obtained for thermal antinociception (plantar test) in naive animals without observable CB_1_-mediated side effects [30].

Protective effects of prophylactic AM1710 (3.2 mg/kg/day s.c.) lasted 17–18 days following drug removal and outlasted the anti-allodynic effects of WIN55,212-2. Unlike WIN55,212-2, anti-allodynic effects of AM1710 (3.2 mg/kg/day s.c.) were mediated by CB_2_ activation only. Our lab previously demonstrated AM1710-mediated amelioration of allodynia during the maintenance phase of paclitaxel-induced nociception (day 20) following acute administration [15]. Four CB_2_ agonists have been shown to ameliorate established paclitaxel-induced neuropathic nociception [14,15,31,32]. Notably, repeated administration of the CB_2_ agonist MDA7 (15 mg/kg i.p. × 14 days) 15 min prior to behavioral evaluations also blocked paclitaxel-induced mechanical allodynia through a CB_2_ mechanism during the phase of drug delivery (i.e., behavioral effects blocked by AM630 and absent in CB_2_^-/-^ mice) [16].

Long-term alterations in lumbar spinal cord CB_1_ or CB_2_ mRNA expression (day 22) were not induced by paclitaxel- relative to cremophor-treatment in our study by RT-PCR. However, prophylactic AM1710 (3.2 mg/kg/day s.c.) increased both CB_1_ and CB_2_ mRNA expression, an effect blocked by concurrent AM630 (3 mg/kg/day s.c.) administration. By contrast, MDA7 (15 mg/kg i.p. × 14 days) has been reported to normalize paclitaxel-induced increases in lumbar spinal cord CB_2_ receptor protein [16]. Differences in paclitaxel dosing (cumulative dose: 8 vs. 4 mg/kg i.p.), CB_2_ agonist (AM1710 vs. MDA7), route of administration (mini pump vs. once-daily injections), or protein vs. mRNA analysis could help account for these differential findings.

### Lack of side effects after either WIN55,212-2 or AM1710 treatment

In our study, animals remained in good health and showed either normal or enhanced weight gain. WIN55,212-2 (0.1 mg/kg/day s.c.) increased weight gain in paclitaxel-treated rats. CB_1_ activation can produce both orexigenic and metabolic effects to promote weight gain [33]. Interestingly, higher doses of WIN55,212-2 (1–2 mg/kg/day i.p.) failed to attenuate anorexia or weight loss in animals treated with cisplatin [34].

Activity meter assessments conducted during prophylactic treatment (day 19) and following drug removal (day 31), failed to reveal major differences between groups. Thus, chronic infusion of either the mixed CB_1_/CB_2_ agonist or the CB_2_ agonist was unlikely to nonselectively activate CB_1_ receptors; no evidence for hypoactivity [35], a cardinal sign of CB_1_ activation, was observed. These findings are consistent with the results documenting absence of cardinal signs of CB_1_ receptor activation following acute administration of AM1710 [30].

### Potential mechanisms of action for cannabinoid-mediated suppression of paclitaxel-induced neuropathic nociception

Glial activation mediates alterations in synaptic transmission for a number of excitatory and inhibitory mediators known to be important for the maintenance of neuropathic pain states [36]. Because of the prolonged suppression of paclitaxel-induced neuropathy after removal of cannabinoid agonists, we chose to analyze transcriptional changes in markers of glial activation. GFAP mRNA expression in lumbar spinal cord on day 22 (i.e., approximately 24 h after the pump ceased to release drug) showed a trend toward increased expression in paclitaxel- relative to cremophor-treated controls, while no change in CD11b expression was observed. Increases in astrocytic activation (GFAP) with no corresponding changes in microglial activation (OX42, Iba1, and phosphorylated p38) were also recently observed with the same paclitaxel-induced neuropathy dosing protocol used here [12]. In another study, paclitaxel failed to produce microglial activation (% of cremophor-control staining) on day 27 post-treatment [37]. By contrast, MDA7 and WIN55,212-2 suppressed paclitaxel-induced glial activation (on days 28 and 29 post-treatment, respectively) when immunohistochemical staining for astrocytes (GFAP) and microglia (CD11b) was compared with naive animals [11], and it remains unclear whether vehicle or cremophor administration alters glial activation [16]. Cremophor can produce side effects in both clinical use and animal models [38], and assumptions that it is inert are not appropriate.

Prophylactic treatment with either a mixed CB_1_/CB_2_ agonist or a CB_2_ agonist, while failing to produce robust alterations in lumbar spinal cord glial expression, increased CB_1_ and CB_2_ mRNA expression. This effect was blocked by CB_2_ receptor blockade. Upregulation of endocannabinoids and cannabinoid receptors is associated with several neuropathic pain models (for review see [39]). However, to our knowledge, very few, if any, studies have evaluated alterations following prophylactic cannabinoid treatment. Increased receptor densities could increase the potency or efficacy of prophylactic cannabinoids in this model. More work is necessary to determine whether changes in CB_1_ and CB_2_ mRNA levels observed here are also associated with changes in receptor protein. Alternatively, increased CB_1_ and CB_2_ mRNA expression could reflect compensatory changes in transcription following chronic agonist-induced downregulation of receptors. More work is necessary to fully characterize the duration of these effects and their therapeutic implications.

### Translation to the clinic

Our preclinical studies [14,15,17,18] motivated completion of a pilot clinical trial utilizing Sativex, an oromucosal extract containing Δ^9^-tetrahydrocannabinol and cannabidiol, for treatment of chemotherapy-induced neuropathy. Sativex suppressed established chemotherapy-induced neuropathic pain in a subset of responders (5 of 18) in this double-blind placebo-controlled crossover pilot [40], supporting a further evaluation of the clinical viability of cannabinoid-based pharmacotherapy.

## Conclusion

Prophylactic treatment has been tested as a preventive strategy for paclitaxel-induced neuropathic nociception with multiple drugs (for review see [41]). Here, we demonstrate that cannabinoid agonists with different mechanisms of action prevent development of paclitaxel-induced neuropathic nociception during treatment and approximately two to three weeks following cessation of drug delivery. Paclitaxel treatment marginally altered long-term GFAP mRNA expression in lumbar spinal cord and this expression was unaffected by prophylactic cannabinoids, whereas CD11b mRNA expression was unchanged. Prophylactic treatment with either WIN55,212-2 (0.5 mg/kg/day s.c.) or AM1710 (3.2 mg/kg/day s.c.) in paclitaxel animals did, however, increase both CB_1_ and CB_2_ receptor mRNA expression, an effect blocked by concurrent AM630 (3 mg/kg/day s.c.) administration. Some inroads have been made toward discovering mechanisms for cannabinoid-mediated suppression of paclitaxel-induced neuropathy, but more work is necessary to determine the scope and time course of this complex interaction. Our study suggests that further clinical cannabinoid trials [40] for chemotherapy-induced peripheral neuropathy are warranted.

## Methods

### Subjects

#### Rats

One hundred seventy-six adult male Sprague–Dawley rats (beginning weight: 300-400 g; Harlan, Indianapolis, IN) were used in these experiments. All procedures were approved by the University of Georgia Animal Care and Use Committee and followed the guidelines for the treatment of animals of the International Association for the Study of Pain. Animal experiments were conducted in full compliance with local, national, ethical and regulatory principles, and local licensing regulations of the Association for Assessment and Accreditation of Laboratory Animal Care (AAALAC) International’s expectations for animal care and use/ethics committees.

Animals were allowed a minimum of one week habituation prior to beginning the study. Animals were single housed and maintained in a temperature (70-72 °F ± 4 °F) and humidity (30-70%) controlled facility on a 12 hour light cycle (lights on: 07:00 and lights off: 19:00). Food and water was available ad libitum. Following the initial pilot study (n = 17), all animals with osmotic mini pumps were allowed nyla bones (BioServe; Frenchtown, NJ) due to the study duration. Corn cob bedding containing metabolized paclitaxel was treated as chemical hazard waste and disposed of according to appropriate institutional guidelines.

### Drugs and chemicals

Paclitaxel (Taxol) was obtained from Tecoland (Edison, NJ). Polyethylene Glycol 400 (PEG 400) was purchased from VWR International (West Chester, PA). Acetone was purchased from J.T. Baker (Phillipsburg, NJ). Cremophor EL, Dimethyl Sulfoxide (DMSO), and WIN55,212-2 ((*R*)-(+)-[2,3-Dihydro-5-methyl-3[(4-morpholinyl)methyl]pyrrolo[1,2,3-de]-1,4-benzoxazinyl]-(1-naphthalenyl)methanone mesylate salt) were obtained from Sigma Aldrich (St. Louis, MO). AM1710 (3-(1’,1’-dimethylheptyl)-1-hydroxy-9-methoxy-6*H*-benzo[*c*]chromene-6-one), AM251 (*N*-(Piperidin-1-yl)-5-(4-iodophenyl)-1-(2,4-dichlorophenyl)-4-methyl-1*H*-pyrazole-3-carboxamide), and AM630 (6-Iodo-2-methyl-1-[2-(4-morpholinyl)ethyl]-1*H*-indol-3-yl](4-methoxyphenyl)methanone (Iodopravadoline) were synthesized in the Center for Drug Discovery by one of the authors (by GT, VKV, and AZ respectively). Rat subjects received paclitaxel dissolved as previously described [42], administered in a volume of 1 ml/kg. Briefly, paclitaxel was dissolved in a 1:2 ratio of working stock (1:1 ratio of cremophor EL and 95% ethanol) to saline. AM1710, WIN55,212-2, AM251, and AM630 were dissolved in a vehicle of DMSO:PEG 400 in a 1:1 ratio. The selected vehicle was the most compatible for dissolving cannabinoids to be used in Alzet osmotic mini pumps with no reported adverse side effects [43-45].

### General experimental methods

In an initial study, animals were evaluated for development of paclitaxel-induced behavioral sensitization to mechanical and heat stimulation. Responsiveness to different modalities of cutaneous stimulation was assessed on alternate days to avoid sensitization. All subsequent studies used animals surgically implanted with osmotic mini pumps. Baseline assessments of withdrawal thresholds to mechanical and cold (acetone drops) stimulation of the hind paw occurred 48 h (day -8) and 24 h (day -7) prior to surgery, respectively. Osmotic mini pumps (Alzet model 2ML4, Cupertino, CA) were implanted subcutaneously through an incision between the scapulae. Responsiveness to mechanical and cold stimulation was reassessed post-surgery (i.e., after pump implantation but within 48 h prior to initiation (on day 0) of paclitaxel dosing). Animals were weighed on all testing and surgical/sacrifice dates. A subset of animals was sacrificed via live decapitation (day 22) to extract lumbar spinal cords. Certain groups (e.g., antagonist alone conditions, submaximal doses of agonists, cremophor-agonist groups) were only tested through day 22. Osmotic mini pumps were removed in all remaining animals (day 22), and following a short recovery period, responses to mechanical and cold stimulation were reassessed until day 51 post-paclitaxel.

Drug doses were estimated based on the peak osmotic mini pump performance reported by the manufacturer (2.5 μl/hr) and an average rat weight of 375 grams. A small percentage of animals (4.2%) presented with edema around the pump site (seromas). Alzet reports this side effect in < 5% of animals. Treatment for these animals was supervised by the attending veterinarian and consisted of draining fluid every 3 days, or as needed. Six animals (3.6%) were re-sutured following surgery. One of the six animals developed an infection and was treated (from days 16–22) with daily injections of an antibiotic (Enrofloxacin 4.5 mg/ml, 0.4 cc s.c., 2× daily) and sterile water (1 cc s.c., 1× daily) as prescribed by the staff veterinarian. One animal died during the first paclitaxel injection and this animal was excluded from all analyses.

Behavioral measurements, surgeries, chemotherapeutic treatment, and tissue removal were performed by a single experimenter (EJR). Coded testing sheets were used to preserve blinding. Behavioral testing was performed in the presence of a white noise generator to mask extraneous noise.

### Surgical implantation and removal of osmotic mini pumps

Osmotic mini pumps were implanted under isoflurane anesthesia (Isoflo®, Abbott Laboratories, Chicago, IL). The osmotic mini pump was inserted through a surgical incision made between the scapulae; incisions were sutured closed. In the instances where two pumps were implanted (i.e., agonist and antagonist co-administration conditions), pumps were placed in the same pocket. The Alzet model 2ML4 pump has an approximate 2 ml reservoir that releases a preloaded drug or vehicle at a rate of 2.5 ul/hr for approximately 28 days. The pump begins to release the preloaded drug approximately 4–6 hours after implantation; the flow rate is not subject to variations in body temperature. Osmotic mini pumps were weighed before and after being filled with drug or vehicle. The difference of these two values provided an approximate pump fill volume. The animals were given three days (days -5 through -3) to recover from surgery before testing resumed. Animals were either sacrificed or underwent surgery on day 22 to remove pumps; this time point corresponds to the 29th day following pump implantation, at which point the pump should have released its contents. Following pump removal, the residual pump volume was estimated by withdrawing the remaining fluid within the pump reservoir. Animals that underwent surgical removal of osmotic mini pumps were allowed three days of recovery (days 23–25) prior to resumption of behavioral testing.

### Induction of paclitaxel-induced neuropathic nociception

Rats received four once daily intraperitoneal (i.p.) injections of either paclitaxel (2 mg/kg/day i.p.; cumulative dose of 8 mg/kg i.p.) or cremophor: ethanol: saline vehicle (1 ml/kg/day i.p.), administered on alternate days (days 0, 2, 4, and 6) as described previously [28]. Behavioral testing was always performed prior to paclitaxel/vehicle administration.

### Assessment of paw withdrawal latencies to heat stimulation

Paw withdrawal latencies to radiant heat were measured in duplicate for each paw using the Hargreaves test [46] and a commercially available plantar stimulation unit (IITC model 336; Woodland Hills, CA). Rats were placed underneath inverted plastic cages positioned on an elevated glass platform and allowed a minimum of 20 min to habituate prior to testing. Radiant heat was presented to the hind paw midplantar region through the floor of the glass platform. The intensity of the heat source was adjusted such that an average baseline latency of approximately 20 sec was achieved [47]. Stimulation was terminated upon paw withdrawal or after 40 s to prevent tissue damage. Approximately 4 minute interstimulation intervals were allowed between tests. Thermal withdrawal latencies were evaluated before (day 0) and on days 2, 6, 10, 14 and 18 following initiation of paclitaxel dosing. The same animals were tested for the development of mechanical allodynia. Baseline responses to mechanical stimulation (methodology below) were measured (on day 0) before baseline responses to thermal stimulation. A minimum of 1 hour was allowed to elapse between baseline measurements.

### Assessment of mechanical withdrawal thresholds

Mechanical withdrawal thresholds were assessed using a digital Electrovonfrey Anesthesiometer (IITC model Alemo 2390–5; Woodland Hills, CA) equipped with a rigid tip. Rats were placed underneath inverted plastic cages positioned on an elevated mesh platform and allowed a 20 min habituation period prior to testing. Stimulation was applied to the hind paw midplantar region through the floor of a mesh platform. Mechanical stimulation was terminated upon paw withdrawal; consequently, no upper threshold limit was set for termination of a trial. Two thresholds were taken for each paw. Approximately 2 minute interstimulation intervals were allowed between tests. Mechanical withdrawal thresholds were measured on days 0, 4, 8, 12, 16 and 20 for animals that did not receive osmotic mini pumps (Figure 1). Mechanical withdrawal thresholds were measured every 2–6 days (i.e., days -8, -2, 0, 2, 4, 6, 8, 10, 12, 14, 16, 18, and 20) for all animals that received osmotic mini pumps. A subset of osmotic mini pump animals were tested until day 50 (testing continued with the following schedule: days 26, 28, 30, 32, 34, 36, 38, 40, 42, 44, 46, 48, and 50).

### Assessment of cold allodynia

Cold allodynia was assessed using acetone drops applied to the hind paw midplantar surface as previously described [15,48]. Rats were placed underneath inverted plastic cages positioned on an elevated mesh platform and allowed a 20 min habituation period prior to testing. Acetone was loaded into a one cc syringe barrel with no needle tip. One drop of acetone (approximately 20 μl) was applied through the mesh platform onto the hind paw midplantar surface. Care was taken to gently apply the bubble of acetone to the skin without inducing mechanical stimulation by syringe barrel contact with the paw.

Paw withdrawal was recorded as a binary response (presence or absence) and was frequently accompanied by nocifensive behaviors (e.g., rapid flicking of the paw, chattering, biting, and/or licking of the paw). These nocifensive behaviors were recorded as duration of acetone response. Five measurements were taken for each paw. Testing order alternated between paws (i.e., right, left). No cut-off latency was enforced. Approximately 2 min interstimulation intervals were allowed between testing of right and left paws. A minimum interstimulation interval of 5 min was allowed between testing each pair of paws (right and left). Cold allodynia testing took place on days -7, -1, 5, 11, 17 and 21 for all animals with osmotic mini pumps. Five days were allowed between assessments of cold allodynia to avoid hypersensitivity with one exception. Animals were tested on day 21 because osmotic mini pumps would purportedly still be releasing drug (i.e., 28 days following pump implantation). A subset of animals was tested to day 51 (i.e., testing for these animals continued with the following schedule: days 27, 33, 39, 45, and 51).

### Locomotor activity

Total distance traveled (cm) was assessed using an activity monitor chamber (Coulbourn Instruments, Whitehall, PA) measuring 40.64 cm^3^. The apparatus was housed in a darkened room and red light was used to provide illumination. Tracking beams were positioned 2.54 cm apart giving 1.27 cm in spatial resolution. Activity was automatically measured by computerized analysis of photobeam interrupts (TruScan 2.0; Coulbourn Instruments, Whitehall, PA). Animals were allowed a minimum of 15 minutes to habituate to the room prior to being placed undisturbed in the activity meter for 15 min. Chlorhexidine was used to clean the activity meter after each animal. Activity meter assessment took place both during (day 19) and following termination (day 31) of drug delivery in a subset of animals that received chronic infusions.

### Prophylactic drug groups

Animals were randomly assigned to drug treatments. Animals assigned to the paclitaxel condition received pumps filled with the mixed CB_1_/CB_2_ agonist WIN55,212-2 (1, 0.5, or 0.1 mg/kg/day s.c., n = 8–10 per group), the CB_2_-preferring agonist AM1710 (3.2, 0.32, or 0.032 mg/kg/day s.c., n = 8–14 per group), vehicle (DMSO:PEG 400, n = 14), or saline (n = 4). Animals assigned to the cremophor-vehicle control condition received pumps filled with either WIN55,212-2 (0.5 mg/kg/day s.c., n = 8), AM1710 (3.2 mg/kg/day s.c., n = 8), vehicle (DMSO:PEG 400, n = 10), or saline (n = 4).

Pharmacological specificity was assessed in paclitaxel-treated animals implanted concurrently with two osmotic mini pumps. One pump contained an antagonist (either AM251 (3 mg/kg/day s.c.) or AM630 (3 mg/kg/day s.c.)) and the other pump contained an agonist (either WIN55,212-2 (0.5 mg/kg/day s.c., n = 10 per group) or AM1710 (3.2 mg/kg/day s.c., n = 10 per group)). Separate groups of paclitaxel-treated animals received pumps filled with either AM251 (3 mg/kg/day s.c., n = 8) or AM630 (3 mg/kg/day s.c., n = 8).

### Quantification of lumbar spinal cord mRNA

Real time RT-PCR was used to quantify mRNA levels in lumbar spinal cords removed from animals sacrificed on day 22. Methods are described previously [49]. RNA from paclitaxel-treated animals that received vehicle, WIN55,212-2 (0.5 mg/kg/day), WIN55,212-2 (0.5 mg/kg/day) + AM630 (3 mg/kg/day), AM1710 (3.2 mg/kg/day), AM1710 (3.2 mg/kg/day) + AM630 (3 mg/kg/day), or from cremophor-vehicle-treated animals (n = 4 per group) were extracted using a TRIzol (Invitrogen)/RNeasy (Qiagen) hybrid protocol according to manufacturer’s instructions. Purified RNA from each sample was then treated with DNase 1. Expression levels of GFAP, CD11b, CB_1_, and CB_2_ mRNAs were quantified using one step RT-PCR in a Mastercycler ep realplex RT-PCR machine (Eppendorf North America Inc., Hauppauge, NY) using PowerSYBR green PCR kit (Applied Biosystems, Carlsbad, CA). GAPDH (glyceraldehyde-3-phosphate dehydrogenase) was used as internal standard to normalize mRNA levels. Primers used were as follows: rat GAPDH (sense: 5′-ATGACTCTACCCACGGCAAG-3′, anti-sense: 5′CATACTCTGCACCAGCATCTC-3′); rat GFAP (sense: 5′-GAGTCCACAACCATCCTTCTGAG-3′, anti-sense: 5′-ACACCAGGCTGCTTGAACAC-3′); rat CD11b (sense: 5′-CTGGGAGATGTGAATGGAG-3′, anti-sense: 5′-ACTGATGCTGGCTACTGATG-3′); rat CB_1_ (sense: 5′-CTACTGGTGCTGTGTGTCATC-3′ and anti-sense: 5′-GCTGTCTTTACGGTGGAATAC-3′); rat CB_2_ (sense: 5′-GCAGCCTGCTGCTGACCGCTG-3′, anti-sense: 5′-TGCTTTCCAGAGGACATACCC-3′).

### Statistical analyses

Percentage of paw withdrawals from acetone application to the hind paws was calculated using the following formula: ((Total number of paw withdrawals) * 100)/10. Data were analyzed using analysis of variance (ANOVA) for repeated measures, one-way ANOVA, or planned comparison *t*-test as appropriate. SPSS 19.0 (SPSS Incorporated, Chicago, IL, USA) statistical software was employed. The Greenhouse-Geisser correction was applied to all repeated factors where the epsilon value from Mauchly’s Test of Sphericity was < 0.75 and significance level was *P* < 0.05. Degrees of freedom reported for interaction terms of repeated factors are uncorrected values in cases where the Greenhouse-Geisser correction factor was applied. Post-hoc comparisons between the primary control group (paclitaxel-vehicle) and other experimental groups were performed using the Dunnett test (2-sided). Post-hoc comparisons between different experimental groups were also performed to assess dose–response relationships and pharmacological specificity using the Tukey test. Levene’s test for homoscedasticity was applied to all planned comparison t-tests. *P <* 0.05 was considered statistically significant.

## Abbreviations

CD11b: Cluster of differentiation molecule 11B; GFAP: Glial fibrillary acidic protein; i.p.: Intraperitoneal; PEG 400: polyethylene glycol 400; s.c.: Subcutaneous.

## Competing interests

Dr. Alexandros Makriyannis serves as a consultant for MAK Scientific. No other authors declare competing interests.

## Authors’ contributions

EJR contributed to experimental design, completed all surgeries, behavioral studies, and tissue extractions, analyzed data and drafted the manuscript. LD isolated RNA, carried out the RT-PCR studies and analyzed data. GAT synthesized AM1710. VKV synthesized AM251 and AM630. AMZ synthesized AM630. YYL assisted with RT-PCR and contributed to manuscript preparation and data interpretation. AM provided cannabinoid compounds and contributed to data interpretation. AGH designed the study, participated in its coordination and implementation, and wrote the manuscript with EJR. All authors read and approved the final manuscript.
